# miR-103a-3p and miR-22-5p Are Reliable Reference Genes in Extracellular Vesicles From Cartilage, Adipose Tissue, and Bone Marrow Cells

**DOI:** 10.3389/fbioe.2021.632440

**Published:** 2021-02-15

**Authors:** Enrico Ragni, Alessandra Colombini, Paola De Luca, Francesca Libonati, Marco Viganò, Carlotta Perucca Orfei, Luigi Zagra, Laura de Girolamo

**Affiliations:** ^1^IRCCS Istituto Ortopedico Galeazzi, Laboratorio di Biotecnologie Applicate all’Ortopedia, Milan, Italy; ^2^IRCCS Istituto Ortopedico Galeazzi, Hip Department, Milan, Italy

**Keywords:** cartilage cells, adipose tissue mesenchymal stromal cells, bone marrow mesenchymal stromal cells, miRNAs, reference genes, extracellular vesicles

## Abstract

Cartilage cells (CCs), adipose tissue (ASC)- and bone marrow (BMSC)-derived mesenchymal stromal cells (MSCs) have been shown as promising candidates for the treatment of osteoarthritis (OA). Despite their adaptive ability, exposure to chronic catabolic and inflammatory processes can limit their survival and healing potential. An attractive cell-free alternative or complementary strategy is represented by their secreted extracellular vesicles (EVs), having homeostatic properties on OA chondrocytes and synovial cells. In view of clinical translation, a thorough characterization of the shuttled therapeutic molecules, like miRNAs, is greatly needed to fingerprint and develop the most effective EV formulation for OA treatment. To date, a crucial pitfall is given by the lack of EV-miRNA-associated reference genes (RGs) for the reliable quantification and comparison among different therapeutic EV-based therapeutic products. In this study, the stability of 12 putative miRNA RGs (let-7a-5p, miR-16-5p, miR-22-5p, miR-23a-3p, miR-26a-5p, miR-29a-5p, miR-101-3p, miR-103a-3p, miR-221-3p, miR-423-5p, miR-425-5p and miR-660-5p), already proposed by literature in EV products from alternative sources, was assessed in EVs isolated from three donor-matched ASCs, BMSCs, and CCs through geNorm, NormFinder, BestKeeper, and ΔCt algorithms and the geometric mean of rankings. ASC-EVs and BMSC-EVs shared more similar molecular signatures than cartilage-derived EVs, although overall miR-103a-3p consistently ranked as the first and miR-22-5p as the second most stable EV-miRNA RG, whereas miR-221-3p behaved poorly. Further, to emphasize the impact of incorrect RG choice, the abundance of four OA-therapeutic miRNAs (miR-93-5p, miR-125b-5p, miR-455-3p, and miR-27b-3p) was compared. The use of miR-221-3p led to less accurate EV fingerprinting and, when applied to sift therapeutic potency prediction, to misleading indication of the most appropriate clinical product. In conclusion, miR-103a-3p and miR-22-5p will represent reliable RGs for the quantification of miRNAs embedded in MSC- and CC-EVs, a mandatory step for the molecular definition and comparison of the clinical potency of these innovative cell-free-based therapeutic products for OA in particular, as well as for a wider array of regenerative-medicine-based approaches.

## Introduction

Articular cartilage cells (CCs) (Brittberg et al., 1994; Schuette et al., 2017; Kreuz and Kalkreuth, 2019), bone marrow- (BMSCs) and adipose tissue-derived stromal cells (ASCs) (Moroni and Fornasari, 2013) are considered the most clinically relevant cell types in the setting of cell-based therapy for the treatment of osteoarthritis (OA). The adaptive trophic and immunomodulatory potential of CCs, ASCs, and BMSCs along with the crosstalk with the resident and inflammatory infiltrated cells account for their capacity to actively modulate the local pathological environment (Colombini et al., 2019).

This was confirmed by the satisfactory outcomes in terms of pain relief and joint homeostasis restoration in osteoarthritic patients (Niemeyer et al., 2016; Colombini et al., 2019; Kim et al., 2019). However, the typical chronic catabolic and inflammatory processes of an OA joint represents, together with the activation trigger, a big hurdle to cell survival and healing performances (Colombini et al., 2019). Moreover, the achievement of a sufficient number of cells for autologous use is associated with expensive and time-consuming procedures, together with patients’ discomfort (Lopa et al., 2019).

The switch toward allogeneic donors could be a cheaper and logistically more convenient solution for the MSC-based treatment of musculoskeletal conditions (Vega et al., 2015; Gupta et al., 2016). More importantly, the collection and characterization of several cell batches from healthy donors would allow us to select only those with the best potency. Likewise, allogeneic CCs have been demonstrated to possess potent immunomodulatory properties (Lohan et al., 2016) and similar efficacy in a rabbit model of cartilage defect when compared to autologous CCs (Boopalan et al., 2019). Nevertheless, the potential tumorigenicity (Barkholt et al., 2013) and pro-fibrogenic potential (Russo et al., 2006) of autologous or allogeneic MSCs and the production of allo-antibodies (Cho et al., 2008) or the host immune response mediated by allogeneic cells (Moskalewski et al., 2002) still remain a concern.

Waiting for the development and validation of safe and clinically effective allogeneic cell therapies for OA treatment, an attractive alternative, or a strategy to combine with autologous cells is represented by the use of non-immunogenic cell products such as a conditioned medium and/or extracellular vesicles (EVs) (Colombini et al., 2019; Yin et al., 2019; Wu et al., 2020). In particular, the conditioned medium from BMSCs (van Buul et al., 2012) or ASCs (Manferdini et al., 2013; Platas et al., 2013, 2016) had anti-inflammatory and homeostatic properties in inflamed or OA chondrocytes and synovial cells, mostly mediated by their EVs. When co-cultured with osteoarthritic CCs, human BMSC-EVs were able to stimulate the production of proteoglycans and type II collagen from CCs and to inhibit the catabolic effects induced by pro-inflammatory mediators (Vonk et al., 2018). In addition, EVs derived from BMSCs promoted cartilage tissue formation by enhancing proliferation and attenuating apoptosis of CCs (Cosenza et al., 2017; Toh et al., 2017; Zhang et al., 2018). EVs mediated also the ASC anti-inflammatory and chondro-protective action in osteoarthritic CCs stimulated with IL-1β (Tofiño-Vian et al., 2018). CCs treated with ASC-EVs showed a downregulation of COX-2, PGES-1, and iNOS expression, with a consequent reduction of the release of TNF-α, IL-6, PGE2, and NO and a reduced MMP activity and MMP13 expression. Moreover, the enhancement of the IL-10 release and the type II collagen expression suggested a trophic activity of ASC-EVs. Similar results were reported also for synovial fibroblasts isolated from osteoarthritic joints (Ragni et al., 2019c). Regarding CCs, recent findings showed that also their EVs stimulated chondrogenesis and were chondro-protective, by decreasing catabolic events in IL−1β−treated CCs (Liu et al., 2020). Moreover, *in vitro*, CC-EVs were able to stimulate proliferation, migration, and expression of chondrogenesis markers in constructs containing cartilage progenitor cells, while inhibiting angiogenesis (Chen et al., 2018). *In vivo*, CC-EVs stabilized these constructs, which efficiently and reproducibly developed into cartilage with increased collagen deposition, minimal hypertrophy, and vessel ingrowth. On the contrary, cartilage formed with BMSC-EVs was characterized by hypertrophic differentiation accompanied by vascular ingrowth (Chen et al., 2018). Therefore, CCs could also be exploited to produce EVs, possibly even more committed to cartilage healing and homeostasis. This rationale relies on the nature of the EV cargo that is cell type specific and resembles the properties of the secreting cells. Consistently, when compared with ASCs or BMSCs, CCs derived from OA donors showed higher chondrogenic ability, superior basal secretion of growth factors and cytokines, and noteworthy immunomodulatory behavior (De Luca et al., 2019).

In this scenario, the use of EVs has the considerable advantage of being potentially well characterized in terms of their contents at the molecular level, including lipids, proteins, or miRNAs. In the last years, miRNA fingerprint in EVs from several cell types, including MSCs (Ferguson et al., 2018; Qiu et al., 2018), has started to be deciphered, shedding light on shuttled biological functions and providing a platform for dissecting the overall EV potential. Therefore, a deep characterization and comparison of EVs from different cell types in terms of miRNA content would allow the selection of the most effective drug-like formulation to be used as a therapeutic tool in each specific pathology, including OA. In this perspective, specific EV-miRNA engineering (Tao et al., 2017) or modifications of their natural amounts due to variable secreting cells or stage of differentiation (Wang et al., 2018) were postulated as a next-generation approach. Nevertheless, to achieve a complete miRNA characterization, high-throughput quantitative miRNA expression analysis should be performed. Global mean normalization (Mestdagh et al., 2009) is the more reliable strategy to compare outcomes, although it implies the need of a large amount of collected EVs due to the very low nucleic acid amount per particle. While this is possible for basic research, in the therapeutic pipeline, this would be time-consuming and non-cost-effective. Therefore, reliable reference genes (RGs) would be needed to both validate high-throughput data and establish focused assays to characterize different batches. Nevertheless, to date, a universal miRNA RG has not been defined yet, and each EV type or experimental setting must be investigated (D’Haene et al., 2012). The purpose of the present study was to characterize the stability of putative EV-miRNA RGs in donor-matched ASCs, BMSCs, and CCs via bioinformatic tools. The selection of the most stable RGs will allow a reliable comparison of the content of the EVs obtained from three different tissue sources, as well as from different donors.

## Materials and Methods

### Ethics Approval Statement

Institutional review board approval (San Raffaele Hospital Ethics Committee approval on 8 March 2018, registered under number 6/int/2018) was obtained and sampling performed after the procurement of patient informed consent and following the 1964 Helsinki declaration and its later amendments.

### CCs, BMSCs, and ASCs Isolation and Expansion

Articular cartilage, bone marrow, and subcutaneous adipose tissue were collected from the same three osteoartritic patients (two females aged 53 and 56 years and one male aged 41 years, Kellgren–Lawrence III–IV) who underwent total hip arthroplasty. Cartilage was detached with a scalpel from non-weight-bearing superficial areas of femoral head/neck (removed to allow implant positioning) and enzymatically digested (37°C, 22 h) with 0.15% w/v type II collagenase (Worthington Biochemical, Lakewood, NJ, United States) (Lopa et al., 2013). Bone marrow coming out from the femoral canal was collected, washed in phosphate-buffered saline (PBS), and centrifuged, and BMSCs were isolated for plastic adherence (Lopa et al., 2011). A small waste amount of subcutaneous adipose from local hip fat deposit tissue was collected, and ASCs were isolated by enzymatic digestion (37°C, 30 min) with 0.075% w/v type I collagenase (Worthington Biochemical, Lakewood, NJ, United States) (Lopa et al., 2014).

The obtained CCs were cultured in high-glucose (4.5 mg/ml) Dulbecco’s Modified Eagle Medium (DMEM), and ASCs and BMSCs were cultured in minimum essential medium (αMEM). All cells were supplemented with 10% fetal bovine serum (FBS, Lonza), 0.29 mg/ml L-glutamine, 100 U/ml penicillin, 100 μg/ml streptomycin, 10 mM 4-(2-hydroxyethyl)piperazine-1-ethanesulfonic acid (HEPES), and 1 mM sodium pyruvate (all reagents from Thermo Fisher Scientific, Waltham, MA, United States).

To preserve MSC chondrogenic potential, 5 ng/ml of fibroblast growth factor 2 (FGF-2) (PeproTech, Rocky Hill, NJ, United States) was added to BMSCs and ASCs (Solchaga et al., 2005; Liu and Wagner, 2012). All the cell types were cultured at 37°C, 5% CO_2_, and 95% humidity.

Experiments with EVs were performed on cells at passage 3, and flow cytometry was performed at passage 4.

### Cell Characterization

Flow cytometry analysis was performed on 2.5 × 10^5^ cells incubated with anti-human primary monoclonal antibodies: CD14-FITC, CD34-biotinylated, CD44-FITC, CD45-FITC, and CD105-biotinylated (Ancell Corporation, Bayport, MN, United States) and CD90-FITC and CD73-PE (Miltenyi Biotec, Bergisch Gladbach, Germany). Streptavidin–phycoerythrin (PE) (Ancell Corporation, Bayport, MN, United States) was added to biotinylated stained cells. Data were acquired using a FACSCalibur flow cytometer collecting a minimum of 10,000 events and analyzed using CellQuest software (BD Biosciences, San Jose, CA, United States).

### EV Isolation and Nanoparticle Tracking Analysis (NTA)

To obtain the cell supernatants, the cells at 90% confluency were washed three times with PBS, and a medium without FBS was added. After 48 h and cell viability check with a NucleoCounter NC-3000 (ChemoMetec, Allerød, Denmark), 30 ml of culture supernatants was collected and differentially centrifuged at 4°C to remove debris and floating cells at 376 × *g* for 15 min, 1,000 × *g* for 15 min, 2,000 × *g* for 15 min, and twice at 4,000 × *g* for 15 min each (cleared supernatant). Dilutions of 1:3 were performed in PBS, and 1 ml dilution was analyzed with the NanoSight LM10-HS system (NanoSight Ltd., Amesbury, United Kingdom) (Ragni et al., 2017). Five recordings of 60 s were conducted for each EV sample, and an NTA-dedicated software was used to provide both concentration measurements and high-resolution particle size distribution profiles.

### EV Characterization

The cleared supernatants, as previously reported, were ultracentrifuged at 100,000 × *g* for 3 h at 4°C in a 70 Ti rotor (Beckman Coulter). The pellets were suspended in 100 μl of PBS and processed as follows:

(a) Transmission electron microscopy (TEM): 5 μl of EVs in PBS was absorbed on formvar carbon-coated grids for 10 min, and particles were negatively stained with 2% uranyl acetate aqueous suspension for 10 min. Samples were analyzed with a TALOS L120C TEM (Thermo Fisher Scientific, Waltham, MA, United States) at 120 kV.
(b) Flow cytometry: EVs were diluted at 1:150 in PBS, and 0.05 μM carboxyfluorescein succinimidyl ester (CFSE) staining was performed for 30 min at 37°C in the dark. EVs without CFSE were used as a mock sample. Then, 1 μl of antibodies (BioLegend, San Diego, CA, United States) anti-CD9-APC (HI9A), CD63-APC (H5C6), and CD81-APC (5A6) (for EV markers) or CD44-APC, CD73-APC, and CD90-APC (for stomal markers) was added separately to 20 μl of CFSE-EVs, and incubation was performed for 30 min at 4°C in the dark. After a further 1:7 dilution with PBS, samples were analyzed with a CytoFLEX flow cytometer with a 10 μl/min flow rate. At least 30,000 events were acquired. Events were compared with those obtained when running FITC fluorescent beads of 160, 200, 240, and 500 nm (Biocytex, Marseille, France), and PBS or unstained EVs were used to gate FITC-positive events. PBS samples supplemented with CFSE and/or antibodies were used as negative controls for background in FITC and APC channels (Ragni et al., 2020b).

### Candidate miRNA RG Selection

According to the literature, 12 miRNAs were selected for stability analysis (Table 1; Cazzoli et al., 2013; Ge et al., 2014; Santovito et al., 2014; Li et al., 2015a,b; Gouin et al., 2017; Lange et al., 2017; Ragni et al., 2019d).

**TABLE 1:** Candidate RGs and cartilage-related miRNAs and target sequences.

Accession number	Gene name	Target sequence (5’–3’)	References
MIMAT0000062	let-7a-5p	UGAGGUAGUAGGUUGUAUAGUU	Cazzoli et al., 2013; Li et al., 2015a,b
MIMAT0000069	miR-16-5p	UAGCAGCACGUAAAUAUUGGCG	Ge et al., 2014; Lange et al., 2017
MIMAT0004495	miR-22-5p	AGUUCUUCAGUGGCAAGCUUUA	Lange et al., 2017
MIMAT0000078	miR-23a-3p	AUCACAUUGCCAGGGAUUUCC	Gouin et al., 2017
MIMAT0000082	miR-26a-5p	UUCAAGUAAUCCAGGAUAGGCU	Li et al., 2015a; Gouin et al., 2017
MIMAT0004503	miR-29a-5p	ACUGAUUUCUUUUGGUGUUCAG	Ragni et al., 2019d
MIMAT0000099	miR-101-3p	UACAGUACUGUGAUAACUGAA	Gouin et al., 2017
MIMAT0000101	miR-103a-3p	AGCAGCAUUGUACAGGGCUAUGA	Li et al., 2015b
MIMAT0000278	miR-221-3p	AGCUACAUUGUCUGCUGGGUUUC	Li et al., 2015a,b
MIMAT0004748	miR-423-5p	UGAGGGGCAGAGAGCGAGACUUU	Santovito et al., 2014
MIMAT0003393	miR-425-5p	AAUGACACGAUCACUCCCGUUGA	Santovito et al., 2014
MIMAT0003338	miR-660-5p	UACCCAUUGCAUAUCGGAGUUG	Ragni et al., 2019d
**miRNA targets**			
MIMAT0000093	miR-93-5p	CAAAGUGCUGUUCGUGCAGGUAG	Ding et al., 2019; Xue et al., 2019; Wu et al., 2020
MIMAT0000419	miR-27b-3p	UUCACAGUGGCUAAGUUCUGC	Akhtar et al., 2010; Wu et al., 2014, 2020
MIMAT0000423	miR-125b-5p	UCCCUGAGACCCUAACUUGUGA	Matsukawa et al., 2013; Wu et al., 2014, 2020
MIMAT0004784	miR-455-3p	GCAGUCCAUGGGCAUAUACAC	Swingler et al., 2012; Wu et al., 2014, 2020

### RNA Isolation and miRNA Profiling

Five milliliters of cleared culture supernatants containing similar number of EVs (10.6 × 10^9^ EVs ± 1.3) was 1:1 diluted in PBS and ultracentrifuged at 100,000 × *g* for 9 h at 4°C, and the pellet was frozen at -80°C until RNA extraction and miRNA profiling as previously reported (Ragni et al., 2019c). Briefly, before RNA extraction, *Arabidopsis thaliana* ath-miR-159a (30 pg) synthetic miRNA (Life Technologies, Foster City, CA, United States) was spiked in each sample to monitor the efficiency of RNA recovery by miRNeasy and RNeasy cleanup kits (Qiagen, Hilden, Germany), and cDNA synthesis was performed with standard reverse transcription and pre-amplification steps (Cavalleri et al., 2017). miRNA expression analysis with the OpenArray system (Life Technologies) was performed in 384-well OpenArray plates, and miRNAs with C_RT_ values >27 and Amp Score <1.24 were considered as not present, following manufacturer’s instruction^1^. The mean of each sample was used to equalize subtle differences in the amount of starting RNA. The amplification values of the following assays (Life Technologies) were analyzed for stability: hsa-miR-22-5p 002301; hsa-miR-23a-3p 000399; hsa-miR-29a-5p 002447; hsa-miR-221-3p 000524; hsa-miR-423-5p 002340; hsa-miR-16-5p 000391; hsa-miR-26a-5p 000405; hsa-miR-103a-3p 000439; hsa-miR-101-3p 002253; hsa-let-7d-5p 002283; hsa-miR-425-5p 001516; and hsa-miR-660-5p 001515. The following assays for cartilage-related miRNA analysis (Table 1): hsa-miR-93-5p 001090; hsa-miR-125b-5p 000449; hsa-miR-455-3p 002244; and hsa-miR-27b-3p 000409 (Akhtar et al., 2010; Swingler et al., 2012; Matsukawa et al., 2013; Wu et al., 2014, 2020; Ding et al., 2019; Xue et al., 2019).

### Data Analysis

C_RT_ values of ath-miR-159 Ct spike-in were used for the equalization of technical differences during the whole process (Ragni et al., 2019c). geNorm, NormFinder, BestKeeper, and ΔCt method applets (Vandesompele et al., 2002; Andersen et al., 2004; Pfaffl et al., 2004; Silver et al., 2006) were used to assess miRNA RG stability. The geometric mean, hereafter indicated as geomean, of applet rankings was calculated, leading to an eventual consensus stability score.

Principal component analysis (PCA) plot and heat map were generated with the online package ClustVis^2^ (Metsalu and Vilo, 2015). Raw C_RT_ values of miRNA RGs for heat map and cartilage-related miRNA C_RT_ values normalized with both stable miR-103a-3p/miR-22-5p and unstable miR-221-3p for PCA were analyzed. The following settings were used: for PCA, unit variance scaling was applied to rows; SVD with imputation was used to calculate principal components; and X and Y axes showed principal components 1 and 2; for heat map, rows were centered; unit variance scaling was applied to rows; and both rows and columns were clustered using correlation distance and average linkage.

### Statistical Analysis

GraphPad Prism software version 5 (GraphPad, San Diego, CA, United States) was used to perform statistical analyses. Grubb’s test was used to identify and exclude possible outliers. One-sample Student *t*-test with a significance level set at *p* < 0.05 was used when comparing BMSC-EVs and ASC-EVs with the amount of cartilage-related miRNAs with CC-EVs set as 1.

## Results

### CCs, BMSCs, ASCs, and Derived EV Phenotypic Characterization

Flow cytometry analysis was used to confirm the immunophenotype of CCs, BMSCs, and ASCs. Characteristic MSC cell-surface antigens, including CD44, CD73, CD90, and CD105, were expressed, whereas hematoendothelial markers, such as CD14, CD34, and CD45, were not present (Table 2).

**TABLE 2:** Immunophenotype of CCs, BMSCs, and ASCs as a percentage of marker positivity.

	ASCs	BMSCs	CCs
CD14	1.5 ± 0.6	2.6 ± 1.3	6.0 ± 0.3
CD34	0.8 ± 0.4	0.4 ± 0.4	2.2 ± 0.6
CD45	0.5 ± 0.6	0.2 ± 0.2	2.1 ± 1.1
CD44	99.5 ± 0.3	99.7 ± 0.1	96.3 ± 5.7
CD73	99.8 ± 0.2	99.8 ± 0.1	99.8 ± 0.0
CD90	75.2 ± 17.4	48.1 ± 19.8	98.4 ± 0.6
CD105	93.0 ± 1.9	97.3 ± 0.6	92.4 ± 6.0

**FIGURE 1:**
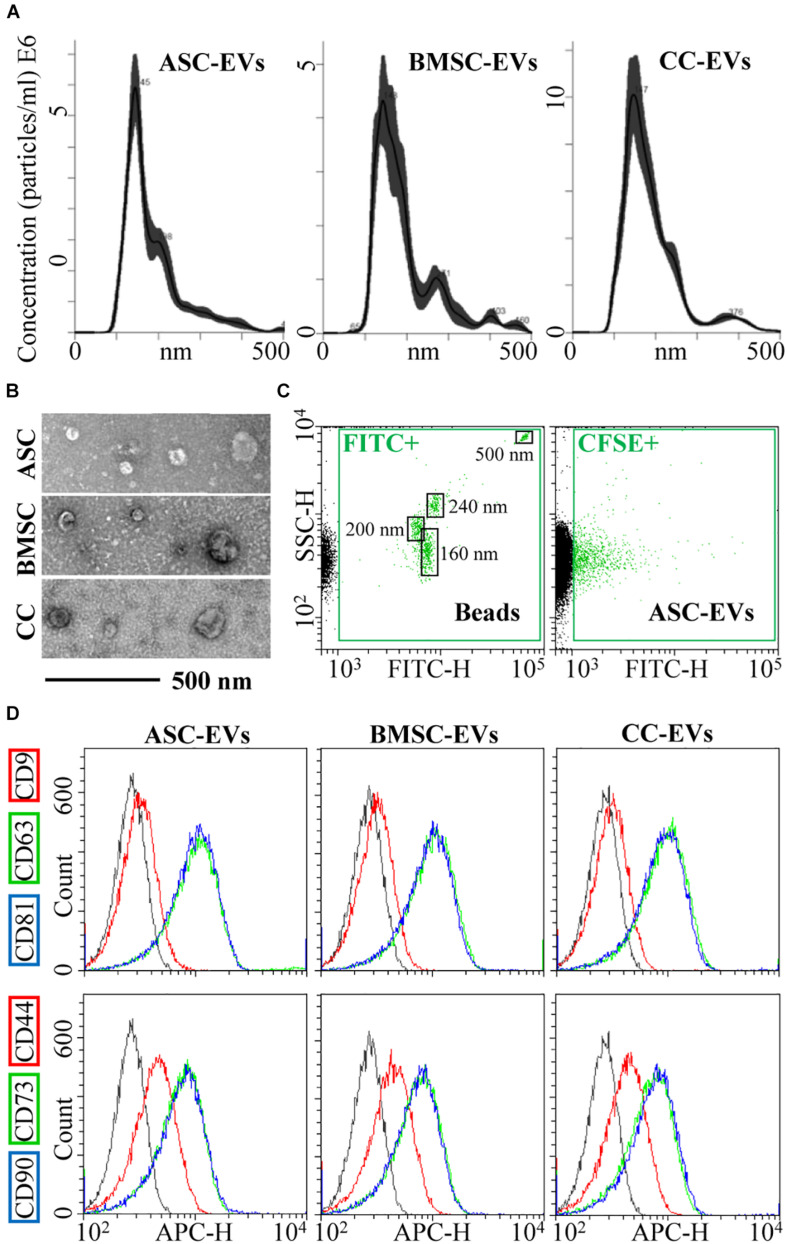
CC-, BMSC-, and ASC-EV phenotype characterization. **(A)** Size distribution of nanoparticles by NanoSight particle tracking analysis. Representative plots are shown. **(B)** TEMs of EVs showing particles with characteristic cup-shaped morphology and size consistent with the NanoSight profile. **(C)** The resolution of the reference bead mix indicates the flow cytometer performance in light scattering at default settings. The first cytogram depicts the SSC-H vs. 535/35 (green fluorescence triggering) channel. Four fluorescent populations (160, 200, 240, and 500 nm) were resolved (FITC+ gate) from the instrument noise (in black). The second plot shows EVs stained with CFSE to allow their identification and gating in the FITC channel (CFSE+ gate) vs. background noise, debris, and unstained particles. **(D)** After CFSE+ gating, with respect to antibody-unstained samples (in gray), antibody-treated EVs showed the presence of EV-defining molecules CD63 and CD81, while CD9 staining gave a weak signal. EVs were also strongly positive for stromal markers CD73 and CD90, with CD44 labeling allowing a complete shift of the population although without a sharp separation with respect to antibody-unstained (gray curve) samples. Representative cytograms are presented.

Cell viability after starvation always resulted to > 95%. EVs released by CCs, BMSCs, and ASCs were directly analyzed in the culture supernatant by NTA. All EVs were within the expected EV size range (mean of 181 nm ± 7 for CCs, 199 nm ± 9 for BMSCs, and 187 nm ± 5 for ASCs) (Figure 1A). D50, the size point below which 50% of the EVs are contained, resulted in 162 nm ± 7 for CCs, 175 nm ± 8 for BMSCs, and 161 nm ± 1 for ASCs, indicating enrichment in small vesicles. After ultracentrifugation, scanning electron microscopy showed the presence of particles within the expected size range, mainly between 50 and 250 nm, with the characteristic cup-shaped morphology (Figure 1B). The dimensional size range was confirmed by flow cytometry by direct comparison with FITC-labeled microbeads of defined size (160–200–240–500 nm) (Figure 1C), and particle integrity was assessed by positive CFSE staining (Figure 1C). Further, all particles strongly expressed both CD63 and CD81, consistent with previously reported characteristics of EVs (Figure 1D). CD9, another EV marker, staining gave a weak signal (Figure 1D). Eventually, stromal markers CD73 and CD90 showed a strong signal in fully and similarly labeled EVs collected from three cell types, while CD44 resulted in a weaker signal, although the complete peak shift suggests a homogeneous staining of the particles rather than two distinct populations (Figure 1D).

### Expression of Candidate RGs

**FIGURE 2:**
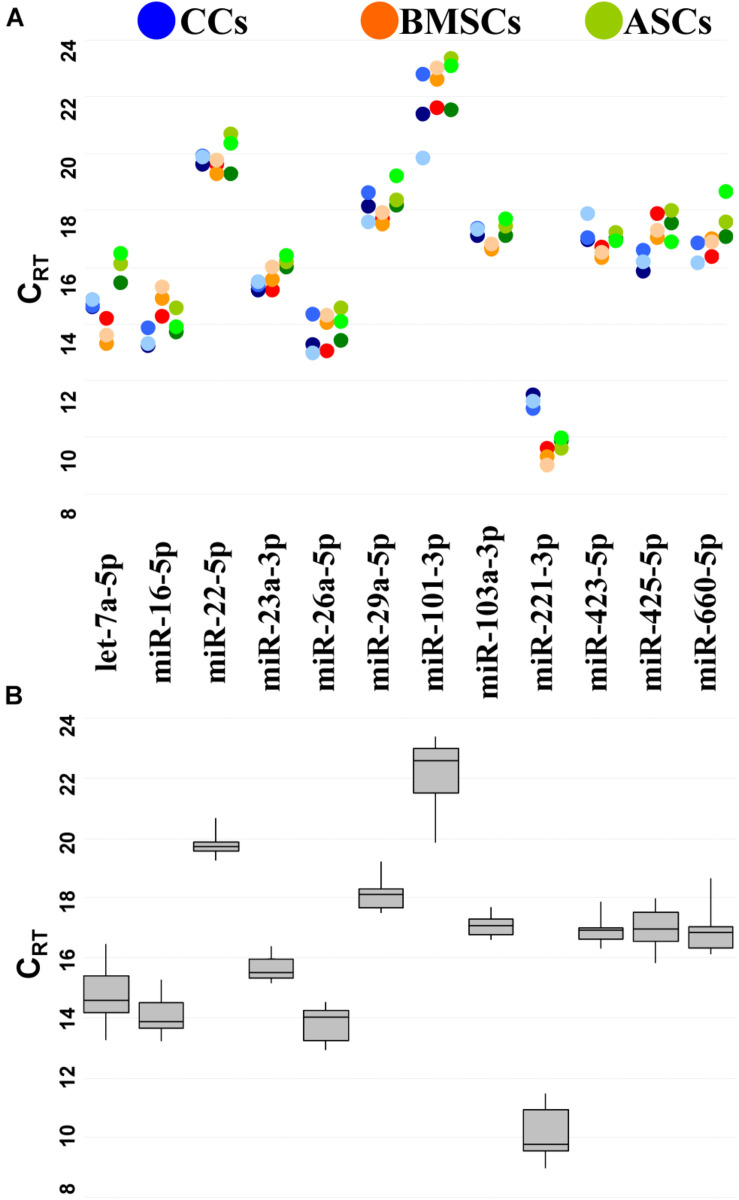
Expression of candidate RG miRNAs in CC-, BMSC-, and ASC-EVs. **(A)** C_RT_ values for miRNA RGs in different donors. Color from dark to light stands for donors 1–3, respectively. **(B)** The box plot graphs of the C_RT_ values for each RG, regardless of the donor difference, illustrate the interquartile range (box) and median. The whisker plot depicts the range of the values.

To reduce likelihood of including co-regulated miRNAs in the stability analysis, miRNA genomic proximity was assessed. None of the 12 candidates resides in the same gene cluster, which makes their levels mutually independent. Then, the presence and amount of each RG were monitored by qRT-PCR. RGs were detected in all the samples at different levels of expression (Figure 2A).

miR-221-3p always had the lowest C_RT_ (high amount), whereas miR-101-3p the highest (Figure 2B). The difference between the most abundant miRNA, miR-221-3p in BMSC3 (C_RT_ 8.99), and the less expressed miRNA, miR-101-3p in ASC2 (C_RT_ 23.35), indicates a range of expression along a 21,000-fold difference for the candidates under analysis. The majority of miRNAs were detected between 14 and 20 C_RT_ values.

### RG Stability Analysis

**FIGURE 3:**
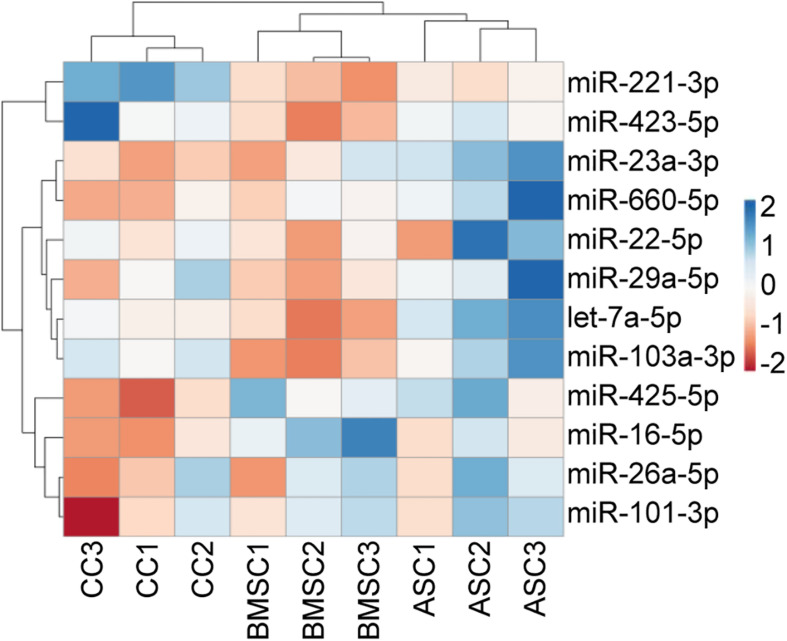
Unsupervised hierarchical clustering for the miRNA RG C_RT_ values. In the dendrogram, each row represents a miRNA, and each column represents a sample. The sample clustering tree is shown at the top. The color scale shown in the map illustrates the relative expression levels of miRNAs across all samples: red shades represent high expression levels (low C_RT_), and blue shades represent low expression levels (high C_RT_).

Before performing the stability analysis of the 12 miRNA RGs under study, unsupervised hierarchical clustering was conducted on the qRT-PCR values. The heat map clearly showed that cell tissue source, and not the donor, tipped the scale toward the identification of similar molecular signatures, with MSCs (BMSCs and ASCs) clustering tighter and under the same node (Figure 3). In particular, with the donors taken into account, MSCs from donors 2 and 3 clustered together, whereas CCs from donor 2 was more similar to those of donor 1.

Following cluster analysis that shed light on the cell source similarity rather than the donor identity, four algorithms (geNorm, NormFinder, BestKeeper, and the comparative ΔCt method) were run, and geometric mean was used to rank the stability of the 12 RGs (Table 3). For all the cell types, miR-103a-3p was the most stable miRNA, whereas miR-101-3p was always ranked last. When MSCs (BMSC-EVs and ASC-EVs) were scored together, again miR-103a-3p was first in the ranking, with let-7a-5p emerging as less reliable and miR-101-3p once more being at the bottom of the list. Then, differences among donors were taken into account (Table 4). As appeared in the hierarchical clustering of the C_RT_ values, donors 1 and 2 were more similar, with miR-26-5p, being the most stable RG, and miR-221-3p in last and penultimate positions, respectively. For donor 3, miR-22-5p was the best RG, and miR-221-3p/miR-101-3p was at the bottom of the ranking. miR-103a-3p always laid in the first half of the list for the three donors, between the fourth and sixth positions.

**TABLE 3:** Stability of candidate RGs in EVs from different cell types.

Source	Ranking	Geomean		Delta Ct		BestKeeper		NormFinder		geNorm	
CCs	1	miR-103a-3p	1.78	miR-22-5p	0.42	miR-23a-3p	0.10	miR-660-5p	0.02	miR-22-5p | miR-103a-3p	0.04
	2	miR-22-5p	2.00	miR-103a-3p	0.42	miR-103a-3p	0.10	miR-16-5p	0.04		
	3	miR-23a-3p	3.46	miR-16-5p	0.43	let-7a-5p	0.11	miR-425-5p	0.11	miR-23a-3p	0.06
	4	miR-16-5p	3.98	miR-23a-3p	0.45	miR-22-5p	0.12	miR-22-5p	0.18	let-7a-5p	0.09
	5	miR-660-5p	4.28	miR-425-5p	0.45	miR-221-3p	0.17	miR-103a-3p	0.19	miR-425-5p	0.18
	6	miR-425-5p	4.61	miR-660-5p	0.46	miR-425-5p	0.25	miR-23a-3p	0.27	miR-16-5p	0.21
	7	let-7a-5p	5.09	let-7a-5p	0.49	miR-16-5p	0.26	miR-29a-5p	0.30	miR-660-5p	0.24
	8	miR-221-3p	7.75	miR-29a-5p	0.57	miR-660-5p	0.32	let-7a-5p	0.36	miR-221-3p	0.30
	9	miR-29a-5p	8.21	miR-221-3p	0.64	miR-29a-5p	0.35	miR-26a-5p	0.47	miR-29a-5p	0.34
	10	miR-26a-5p	9.97	miR-26a-5p	0.66	miR-423-5p	0.40	miR-221-3p	0.51	miR-26a-5p	0.39
	11	miR-423-5p	10.74	miR-423-5p	0.78	miR-26a-5p	0.54	miR-423-5p	0.74	miR-423-5p	0.44
	12	miR-101-3p	12.00	miR-101-3p	1.38	miR-101-3p	1.00	miR-101-3p	1.37	miR-101-3p	0.60
BMSCs	1	miR-103a-3p	1.57	miR-103a-3p	0.38	miR-103a-3p	0.07	miR-29a-5p	0.03	miR-22-5p | miR-29a-5p	0.07
	2	miR-29a-5p	1.57	miR-29a-5p	0.39	miR-423-5p	0.12	miR-103a-3p	0.05		
	3	miR-22-5p	2.45	miR-22-5p	0.41	miR-29a-5p	0.14	miR-22-5p	0.17	miR-103a-3p	0.12
	4	miR-423-5p	3.36	miR-423-5p	0.42	miR-22-5p	0.18	miR-423-5p	0.24	miR-423-5p	0.15
	5	miR-23a-3p	6.12	miR-23a-3p	0.45	miR-221-3p	0.20	miR-23a-3p	0.24	miR-425-5p	0.25
	6	miR-660-5p	6.64	miR-660-5p	0.49	miR-660-5p	0.25	miR-660-5p	0.29	let-7a-5p	0.27
	7	miR-221-3p	6.88	miR-16-5p	0.51	miR-23a-3p	0.28	miR-16-5p	0.40	miR-221-3p	0.30
	8	miR-425-5p	7.54	miR-221-3p	0.58	miR-425-5p	0.33	miR-221-3p	0.48	miR-23a-3p	0.36
	9	miR-16-5p	8.37	miR-425-5p	0.60	let-7a-5p	0.34	miR-425-5p	0.56	miR-660-5p	0.40
	10	let-7a-5p	8.57	let-7a-5p	0.61	miR-16-5p	0.36	let-7a-5p	0.57	miR-16-5p	0.43
	11	miR-26a-5p	11.00	miR-26a-5p	0.64	miR-26a-5p	0.51	miR-26a-5p	0.60	miR-26a-5p	0.48
	12	miR-101-3p	12.00	miR-101-3p	0.67	miR-101-3p	0.53	miR-101-3p	0.65	miR-101-3p	0.51
ASCs	1	miR-103a-3p	1.41	miR-103a-3p	0.43	miR-423-5p	0.11	miR-103a-3p	0.09	miR-23a-3p | miR-103a-3p	0.10
	2	miR-23a-3p	1.68	miR-23a-3p	0.45	miR-23a-3p	0.14	miR-23a-3p	0.19		
	3	miR-423-5p	3.46	let-7a-5p	0.47	miR-221-3p	0.14	let-7a-5p	0.19	miR-221-3p	0.21
	4	let-7a-5p	4.05	miR-26a-5p	0.51	miR-103a-3p	0.21	miR-26a-5p	0.28	miR-423-5p	0.27
	5	miR-221-3p	5.20	miR-16-5p	0.53	miR-16-5p	0.33	miR-16-5p	0.33	let-7a-5p	0.33
	6	miR-16-5p	5.23	miR-423-5p	0.54	let-7a-5p	0.38	miR-423-5p	0.36	miR-16-5p	0.37
	7	miR-26a-5p	5.29	miR-22-5p	0.57	miR-26a-5p	0.39	miR-22-5p	0.41	miR-26a-5p	0.40
	8	miR-22-5p	8.15	miR-29a-5p	0.59	miR-425-5p	0.40	miR-29a-5p	0.46	miR-29a-5p	0.43
	9	miR-29a-5p	8.24	miR-221-3p	0.59	miR-29a-5p	0.42	miR-221-3p	0.46	miR-22-5p	0.45
	10	miR-660-5p	10.24	miR-660-5p	0.72	miR-22-5p	0.55	miR-660-5p	0.64	miR-660-5p	0.49
	11	miR-425-5p	10.84	miR-101-3p	0.76	miR-660-5p	0.59	miR-101-3p	0.67	miR-101-3p	0.53
	12	miR-101-3p	11.24	miR-425-5p	0.82	miR-101-3p	0.75	miR-425-5p	0.76	miR-425-5p	0.58
MSCs*	1	miR-103a-3p	1.41	miR-103a-3p	0.51	miR-423-5p	0.26	miR-103a-3p	0.11	miR-29a-5p | miR-103a-3p	0.23
	2	miR-23a-3p	2.45	miR-23a-3p	0.54	miR-221-3p	0.27	miR-23a-3p	0.15		
	3	miR-423-5p	2.45	miR-423-5p	0.57	miR-23a-3p	0.34	miR-423-5p	0.25	miR-23a-3p	0.26
	4	miR-29a-5p	3.50	miR-22-5p	0.59	miR-103a-3p	0.36	miR-22-5p	0.25	miR-423-5p	0.32
	5	miR-221-3p	4.36	miR-29a-5p	0.59	miR-425-5p	0.38	miR-29a-5p	0.31	miR-221-3p	0.34
	6	miR-22-5p	5.26	miR-221-3p	0.64	miR-29a-5p	0.43	miR-221-3p	0.40	miR-22-5p	0.39
	7	miR-26a-5p	7.24	miR-26a-5p	0.67	miR-26a-5p	0.45	miR-26a-5p	0.46	miR-660-5p	0.43
	8	miR-660-5p	8.18	miR-660-5p	0.71	miR-22-5p	0.46	miR-660-5p	0.50	miR-26a-5p	0.47
	9	miR-425-5p	8.41	miR-101-3p	0.77	miR-16-5p	0.47	miR-101-3p	0.61	miR-101-3p	0.52
	10	miR-101-3p	9.46	miR-425-5p	0.81	miR-660-5p	0.57	miR-425-5p	0.66	miR-425-5p	0.57
	11	miR-16-5p	10.46	miR-16-5p	0.95	miR-101-3p	0.64	miR-16-5p	0.86	miR-16-5p	0.62
	12	let-7a-5p	12.00	let-7a-5p	1.17	let-7a-5p	1.16	let-7a-5p	1.11	let-7a-5p	0.71

Data from three different donors for each cell type were analyzed together to generate a miRNA stability ranking. *RG of BMSCs and ASCs scored together.

**TABLE 4:** Stability of candidate RGs in EVs from single donors.

Source	Ranking	Geomean		ΔCt		BestKeeper		NormFinder		geNorm	
Donor1	1	miR-26a-5p	1.86	miR-26a-5p	0.44	miR-101-3p	0.09	miR-26a-5p	0.03	miR-29a-5p | miR-103a-3p	0.04
	2	miR-423-5p	2.21	miR-423-5p	0.45	miR-423-5p	0.13	miR-423-5p	0.03		
	3	miR-29a-5p	2.91	miR-29a-5p	0.47	miR-26a-5p	0.14	miR-101-3p	0.08	miR-423-5p	0.07
	4	miR-101-3p	2.94	miR-103a-3p	0.48	miR-22-5p	0.15	miR-29a-5p	0.15	miR-26a-5p	0.08
	5	miR-103a-3p	3.16	miR-101-3p	0.50	miR-29a-5p	0.20	miR-103a-3p	0.18	miR-101-3p	0.17
	6	miR-23a-3p	6.24	miR-23a-3p	0.54	miR-103a-3p	0.20	miR-23a-3p	0.29	miR-23a-3p	0.24
	7	miR-22-5p	6.73	miR-660-5p	0.57	miR-16-5p	0.35	miR-660-5p	0.33	miR-660-5p	0.29
	8	miR-660-5p	7.45	miR-22-5p	0.60	miR-23a-3p	0.35	miR-22-5p	0.34	miR-22-5p	0.32
	9	let-7a-5p	9.24	let-7a-5p	0.67	miR-660-5p	0.36	let-7a-5p	0.50	let-7a-5p	0.36
	10	miR-16-5p	9.46	miR-16-5p	0.75	let-7a-5p	0.46	miR-16-5p	0.58	miR-16-5p	0.43
	11	miR-425-5p	11.24	miR-425-5p	1.17	miR-221-3p	0.78	miR-425-5p	1.13	miR-425-5p	0.54
	12	miR-221-3p	11.74	miR-221-3p	1.21	miR-425-5p	0.83	miR-221-3p	1.18	miR-221-3p	0.66
Donor2	1	miR-26a-5p	2.21	miR-101-3p	0.46	miR-26a-5p	0.17	miR-423-5p	0.04	miR-23a-3p | miR-660-5p	0.05
	2	miR-101-3p	2.28	miR-423-5p	0.49	miR-101-3p	0.29	miR-26a-5p	0.08		
	3	miR-660-5p	2.66	miR-26a-5p	0.49	miR-660-5p	0.29	miR-101-3p	0.09	miR-101-3p	0.14
	4	miR-423-5p	2.78	miR-103a-3p	0.50	miR-23a-3p	0.32	miR-103a-3p	0.13	miR-26a-5p	0.20
	5	miR-23a-3p	3.60	miR-660-5p	0.51	miR-103a-3p	0.35	miR-660-5p	0.25	miR-423-5p	0.26
	6	miR-103a-3p	4.68	miR-23a-3p	0.53	miR-423-5p	0.35	miR-22-5p	0.25	miR-103a-3p	0.27
	7	miR-22-5p	7.17	miR-22-5p	0.57	miR-16-5p	0.38	miR-23a-3p	0.28	miR-22-5p	0.31
	8	miR-29a-5p	8.00	miR-29a-5p	0.63	miR-29a-5p	0.43	miR-29a-5p	0.42	miR-29a-5p	0.35
	9	miR-16-5p	9.15	miR-425-5p	0.70	miR-22-5p	0.48	miR-425-5p	0.55	miR-425-5p	0.40
	10	miR-425-5p	9.24	miR-16-5p	0.90	miR-425-5p	0.53	miR-16-5p	0.82	miR-16-5p	0.47
	11	miR-221-3p	11.00	miR-221-3p	1.08	miR-221-3p	0.69	miR-221-3p	1.02	miR-221-3p	0.57
	12	let-7a-5p	12.00	let-7a-5p	1.14	let-7a-5p	0.96	let-7a-5p	1.07	let-7a-5p	0.67
Donor3	1	miR-22-5p	1.68	miR-23a-3p	0.84	miR-22-5p	0.24	miR-22-5p	0.11	miR-26a-5p | miR-425-5p	0.20
	2	miR-23a-3p	2.06	miR-22-5p	0.87	miR-103a-3p	0.31	miR-23a-3p	0.16		
	3	miR-425-5p	3.16	miR-103a-3p	0.93	miR-23a-3p	0.32	miR-29a-5p	0.33	miR-23a-3p	0.35
	4	miR-103a-3p	3.31	miR-29a-5p	0.94	miR-425-5p	0.40	miR-103a-3p	0.36	miR-22-5p	0.46
	5	miR-26a-5p	3.83	miR-425-5p	0.97	miR-423-5p	0.53	miR-425-5p	0.50	miR-103a-3p	0.53
	6	miR-29a-5p	4.74	miR-26a-5p	0.98	miR-26a-5p	0.54	miR-26a-5p	0.53	miR-29a-5p	0.57
	7	miR-423-5p	7.33	miR-660-5p	1.21	miR-29a-5p	0.65	miR-660-5p	0.87	miR-660-5p	0.68
	8	miR-660-5p	7.65	miR-423-5p	1.32	miR-16-5p	0.76	miR-423-5p	1.10	miR-16-5p	0.78
	9	miR-16-5p	8.49	miR-16-5p	1.37	miR-221-3p	0.79	miR-16-5p	1.15	miR-423-5p	0.89
	10	let-7a-5p	10.24	let-7a-5p	1.47	miR-660-5p	0.96	let-7a-5p	1.24	let-7a-5p	0.99
	11	miR-221-3p	10.46	miR-221-3p	1.61	let-7a-5p	1.00	miR-221-3p	1.49	miR-221-3p	1.08
	12	miR-101-3p	12.00	miR-101-3p	1.76	miR-101-3p	1.42	miR-101-3p	1.64	miR-101-3p	1.19

*Data from three different cell types for each donor were analyzed together to generate a miRNA stability ranking.*

Eventually, to obtain a definitive hierarchy, RG stability was calculated regardless of tissue source or donor, analyzing all the samples together (Table 5). miR-103a-3p was ranked first and miR-22-5p second, being in the top 3 positions in all algorithms used. On the contrary, miR-221-3p was at the end of the ranking, being in the last position in three algorithms out of four, making it an unfavorable choice.

**TABLE 5:** Stability of candidate RGs through all samples.

Source	Ranking	Geomean		ΔCt		BestKeeper		NormFinder		GeNorm	
All	1	miR-103a-3p	1.32	miR-103a-3p	0.66	miR-103a-3p	0.29	miR-23a-3p	0.20	miR-22-5p | miR-103a-3p	0.31
	2	miR-22-5p	2.06	miR-23a-3p	0.67	miR-423-5p	0.30	miR-22-5p	0.21		
	3	miR-23a-3p	2.38	miR-22-5p	0.67	miR-22-5p	0.34	miR-103a-3p	0.23	miR-29a-5p	0.37
	4	miR-29a-5p	3.94	miR-29a-5p	0.70	miR-23a-3p	0.39	miR-29a-5p	0.28	miR-23a-3p	0.41
	5	miR-423-5p	5.12	miR-26a-5p	0.75	miR-29a-5p	0.40	miR-26a-5p	0.42	miR-26a-5p	0.48
	6	miR-26a-5p	5.23	miR-660-5p	0.80	miR-26a-5p	0.54	miR-660-5p	0.51	miR-660-5p	0.51
	7	miR-660-5p	6.24	miR-423-5p	0.83	miR-660-5p	0.55	miR-423-5p	0.58	miR-423-5p	0.56
	8	miR-425-5p	8.24	miR-425-5p	0.95	miR-16-5p	0.57	miR-425-5p	0.74	miR-425-5p	0.63
	9	miR-16-5p	8.74	miR-16-5p	0.96	miR-425-5p	0.58	miR-16-5p	0.77	miR-16-5p	0.68
	10	let-7a-5p	10.24	let-7a-5p	1.05	miR-221-3p	0.75	let-7a-5p	0.88	let-7a-5p	0.74
	11	miR-101-3p	11.24	miR-101-3p	1.11	let-7a-5p	0.82	miR-101-3p	0.97	miR-101-3p	0.79
	12	miR-221-3p	11.47	miR-221-3p	1.23	miR-101-3p	0.93	miR-221-3p	1.13	miR-221-3p	0.87

### Impact of RG Choice on the Quantification of Target Genes

Four miRNAs (miR-93-5p, miR-125b-5p, miR-455-3p, and miR-27b-3p), involved in cartilage homeostasis and OA pathology, were studied to evaluate the impact of the RG choice on the accurate expression of these selected miRNAs and on the most reliable establishment of similarities and differences between the three different EV types. Both stable (miR-103a-3p and miR-22-5p) and unreliable (miR-221-3p) RGs were used, with EVs from chondrocytes (CCs) as a touchstone.

**FIGURE 4:**
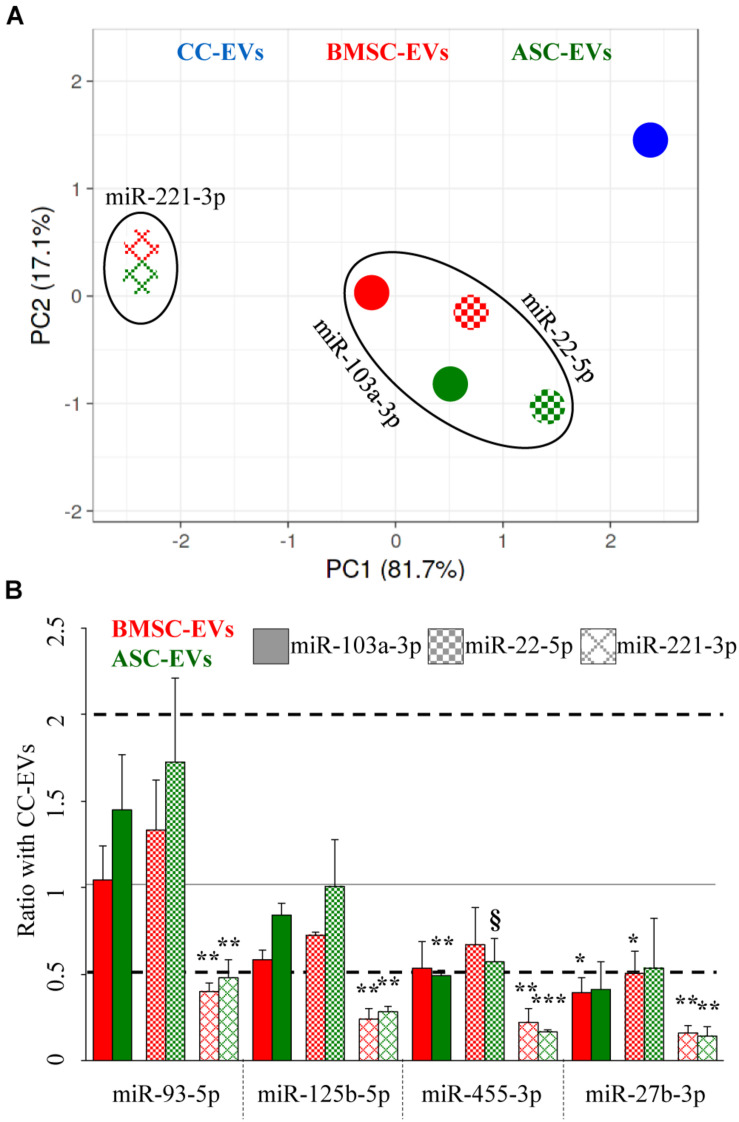
Influence of RG selection on cartilage and OA-related EV-miRNA profile. **(A)** PCA for the BMSC- and ASC-EV molecular signature, based on the amount ratios with CC-EVs set as 1, of cartilage-related miRNAs after stable miR-103a-3p and miR-22-5p or unreliable miR-221-3p RG normalization. **(B)** Effects of RG normalization on the differential expression of single-cartilage-related miRNAs in BMSC- and ASC-EVs with respect to CC-EVs set as 1, *N* = 3, ^§^ *p* < 0.1, *p < 0.05, **p < 0.01, and ***p < 0.001.

Unsupervised clustering analysis of the three EV types was performed, and PCA, with principal components 1 and 2 explaining 99% of the variance, clearly showed that unreliable miR-221-3p led to a sharper separation of BMSC- and ASC-EV samples with respect to CC-EVs (Figure 4A) that, as reported in Figure 3, appeared distinct from MSCs. Further, when using stable miR-103a-3p and miR-22-5p, BMSCs and ASCs grouped close and far from the samples normalized with miR-221-3p, indicating a similar outcome for the two most stable RGs.

Eventually, the differential amount of each candidate miRNAs was analyzed and compared to the value in CC-EVs that resulted in the less clustered sample in the PCA. Notably, the RG choice strongly altered the ratios between samples, exhorting a misleading conclusion (Figure 4B). With reliable RGs, miR-455-3p was downregulated (ratio 0.49, *p*-value 0.0032) in ASC-EVs using miR-103a-3p as RG, with a similar trend for miR-22-5p (ratio 0.57, *p*-value 0.0836). Also, miR-27b-3p got reduced in BMSC-EVs with both RGs. Conversely, when unreliable miR-221-3p was chosen for normalization, all four miRNAs appeared significantly downregulated with respect to CC-EVs. In fact, the mean modulation of the four miRNAs resulted to be 0.64 ± 0.28 and 0.80 ± 0.47 for BMSC- and ASC-EVs using miR-103a-3p, 0.81 ± 0.36 and 0.96 ± 0.55 with miR-22-5p, and 0.26 ± 0.10 and 0.27 ± 0.16 for miR-221-3p. Therefore, the best RG performers obtained through a computational approach allowed us to get closer to the true compositional variation by the reliable evaluation of subtle (around 2-fold) differences between donors.

## Discussion

In this study, among 12 putative stable EV-RGs, miR-103a-3p and miR-22-5p were found to be the most consistent for the reliable quantification of EV-embedded miRNAs released from donor-matched ASCs, BMSCs, and CCs.

EVs are biological delivery platforms used in cell-to-cell communication to shuttle a wide array of molecules, including proteins, lipids, and nucleic acids as miRNAs (Lotvall and Valadi, 2007). Due to their role in regulating physiological and pathological molecular processes (O’Brien et al., 2018), miRNAs have been envisioned as therapeutic molecules (Chakraborty et al., 2017). This paradigm was recently applied to OA (Oliviero et al., 2019), also given the emerging roles of miRNA lack/gain in disease development (Wu et al., 2014, 2020). Therefore, due to their function as a shuttle, EVs were assumed to be a privileged means of miRNA vehicles and therapeutic biological particles for targeted therapies (Raimondo et al., 2019). In this perspective, a reliable normalization approach to compare the presence of potential therapeutic miRNAs between EV batches from different patients or tissue sources is imperative. This issue was recently debated for EVs from umbilical cord-derived MSCs for clinical approaches (Rohde et al., 2019), in order to facilitate translational research during the development and validation of these complex biological therapeutics. Nevertheless, for miRNAs, even when cellular, no univocal RGs have been released, and very often, each sample and each donor have to be tested for the most stable normalizers. To date, only few methods have been proposed. The most sensitive quantification approach is the miRNA global mean expression (Mestdagh et al., 2009). This strategy consists of the obtainment of the entire or at least a large portfolio of miRNome in all samples to be compared and the calculation of the mean expression value for each sample to be used as a normalizer. The major problem is the amount of required RNA that must be abundant enough to obtain the miRNome, especially when qRT-PCR technology is used. Due to the reduced RNA content per EV, approximately 70–25,000 small RNA molecules (Li et al., 2014) with miRNAs accounting only for around 30% of the total, this would imply that a large portion of each EV isolate would be needed for RG identification any time two different batches are compared. Although reasonable for research and preclinical studies aimed at identifying miRNA markers for a specific EV type or disease target, this would make the process economically unsustainable if used for clinical purposes. Thus, in view of translating basic research into potency assays to release disease-targeted EV batches, the availability of few donor/tissue/batch-independent and endogenous RGs is the most straightforward option (Pfaffl, 2001; Meyer et al., 2010; Schwarzenbach et al., 2015). As in cells, also in pure EVs or vesicle-enriched body fluids, U6 snRNA was suggested (Gray et al., 2015; Hayashi et al., 2017). Nevertheless, in recent reports scoring RG stability for EV-miRNAs, U6 snRNA was shown to be an unreliable candidate. In fact, similar to EVs released by cardiosphere-derived-cells (Gouin et al., 2017) and of particular importance for the cell types discussed in this manuscript, U6 snRNA performed poorly also in EVs released by MSCs isolated from adipose tissue (Ragni et al., 2019a,b, d) and amniotic membrane (Ragni et al., 2020a). This may be due to the mechanistical biogenesis of U6 snRNA that is processed by the Drosha complex in spite of the spliceosome (Lee et al., 2003).

Thus, for miRNAs and other small RNAs, it was postulated that miRNA RGs would be more appropriate. In this frame, in the last years, a bunch of reports identified few putative candidates in EVs from an array of different secreting cells (Table 1). Although of pioneering importance, as previously mentioned, a universal miRNA was not defined yet, possibly due to the still reduced number of datasets. In the present study, miR-103a-3p and miR-22-5p were found to be the best overall performers in the comparison of EVs released by CCs, BMSCs, and ASCs. The reported results highlight four crucial findings potentially useful for future comparative studies in the field of EVs:

(i) miR-22-5p was previously identified by our group as reliable EV-miRNA RG for ASCs (Ragni et al., 2019d), naïve or primed with IFNγ, as well as human placenta-derived MSCs (hAMSCs) (Ragni et al., 2020a). Similarly, in ASC-EVs, miR-103a-3p did not change its levels after IFNγ priming (Ragni et al., 2020b), although its absence in hAMSC-EVs (Ragni et al., 2020a) may raise the question of its reliability as a general MSC EV-miRNA RG. Also, both miRNAs were reported as being not influenced in BMSC-EVs by different conditions such as hypoxia or donor age (Zhu et al., 2018; Zhang et al., 2019; Boulestreau et al., 2020). Therefore, the presence of miR-22-5p as a stable miRNA in EVs from different MSC types under several conditions, as well as from CCs, opens the interesting possibility of its use as a reliable EV-miRNA RG to compare several MSCs and most likely any cells of stromal origin. To confirm this hypothesis, future comparative studies focused on miR-22-5p will be needed.
(ii) The tissue source rather than the donor tipped the scale in favor of fingerprint homogeneity (Figure 3). This is of paramount importance, especially in view of clinical translation. In fact, it is reasonable to envision, for allogeneic use, biobanks formed of several EV batches grouped by the original tissue sources rather than by the different donors. Thus, when comparing two or more products with miRNA-based potency assays, e.g., CC-EV batches for cartilage regeneration, the use of cell-type-specific RGs would be of great help.
(iii) The ranking of the tissue sources and the donors differs, with miR-26a-5p being the best in two out of three donors but poor in stability in CCs, BMSCs, ASCs, and MSCs in general (Tables 3, 4). This suggests that in the unlikely, but possible, use of EVs for autologous applications, such as for cutting-edge approaches, where the most effective EV type has to be deciphered from those obtained by two or three tissue sources of the same patient, a different miRNA RG should be selected. miR-26a-5p can be a good starting point for CCs, BMSCs, and ASCs, but its variability between rankings suggests a specific and donor-guided validation.
(iv) Most importantly, even for allogeneic use, it is possible that a combination of tissue sources and donors will have to be screened and compared for the presence of miRNAs targeting a specific disease, if the most effective tissue source has not been defined yet. Only an integrated analysis will give solid outcomes, allowing potency assays to be reliably performed. In this perspective, for CC-, BMSC- and ASC-EVs, miR-103a-3p and miR-22-5p will be a milestone for therapeutic EV-miRNA fingerprint, while when assessing only BMSC- and ASC-EVs miR-103a-3p might be preferred, with miR-22-5p still being among the most stable ones and therefore again suggesting its overall reliability for EVs of mesenchymal origin.

Recently, on a global level, OA cartilage miRNA and mRNA sequencing data allowed the identification of an OA miRNA interactome (Coutinho de Almeida et al., 2019). Several miRNAs were found to regulate crucial pathways; therefore, both their upregulation and downregulation resulted in a key turning point in pathology. This led to the conclusion that EV batches, natural or bio-engineered with protective miRNAs and depleted of the destructive ones, might be more therapeutically relevant, and their comparison, at least for few disease-related candidates, might allow a proper selection. Although covering all the miRNAs related to OA is outside of this study’s scope, we demonstrated the dramatic effect of suboptimal miRNA RG choice on four molecules affecting OA cartilage at different levels, allowing for a sharper definition of their abundance, as close as possible to real levels that, *a priori*, are impossible to know. miR-93-5p is significantly downregulated in the articular cartilages of OA mice, and its overexpression enhances viability, improves apoptosis, and attenuates the inflammatory response in chondrocytes (Ding et al., 2019). Also, miR-125b-5p is downregulated in OA chondrocytes, and its artificial augmentation suppressed the IL-1β-induced upregulation of its target aggrecanase-1 (Matsukawa et al., 2013). On the contrary, miR-455-3p was reported to be highly expressed in human OA cartilage (Swingler et al., 2012). Nevertheless, its upregulation was protective since miR-455-3p promotes TGF-β/Smad signaling inhibiting cartilage degeneration (Hu et al., 2019) and increasing the expression of cartilage-specific genes, and its deletion in mice leads to accelerated cartilage degeneration (Sun et al., 2018). Eventually, already 10 years ago, IL-1β was shown to downregulate miR-27b-3p in human chondrocytes, ending in the increased levels of its target and destructive proteinase MMP-13 (Akhtar et al., 2010). These results thus indicate that the four miRNAs are potential therapeutic agents for the treatment of OA, and their presence/enrichment in therapeutic EVs is a powerful tool. In this frame, the suboptimal choice of RGs, like miR-221-3p, led to an apparent reduction for all analyzed miRNAs in MSCs vs. CCs, erroneously suggesting a reduced therapeutic potential for EVs from BMSCs and ASCs. Future studies on other cartilage- and OA-related miRNAs, and more generally on those miRNAs involved in pathologies that are target of regenerative medicine approaches for a wider clinical translation, will be needed. This will give a more complete picture to compare the presence and/or absence of potentially therapeutic/destructive miRNAs in CC-, BMSC-, and ASC-derived EVs and to develop potency assays for off-the-shelf clinical products.

## Conclusion

EVs from MSCs and CCs have already shown promising results *in vitro* and *in vivo* for the treatment of cartilage-related pathologies like OA. A deep characterization of their content at the molecular level remains a challenge due to the complexity of both cargo and target gene networks. In view of clinical translation, only the definition of reliable RGs will allow a correct quantification of therapeutically relevant embedded molecules, like miRNAs, to develop disease-focused potency assays and compare either cell/tissue sources or donors to make CC-, BMSC-, and ASC-EVs safe and efficacious cell-based miRNA delivery platforms for regenerative medicine-based approaches.

## Data Availability Statement

The datasets presented in this study can be found in online repositories. The names of the repository/repositories and accession number(s) can be found below: https://osf.io/yz6fq/?view_only=e65affa38ae546899bb4112bf4c728f2.

## Ethics Statement

The studies involving human participants were reviewed and approved by the San Raffaele Hospital Ethics Committee approval on date 8 March 2018, registered under number 6/int/2018. The patients/participants provided their written informed consent to participate in this study.

## Author Contributions

AC and ER: conception and design, collection and assembly of the data, data analysis and interpretation, manuscript writing, and final approval of the manuscript. LdG: financial and administrative support and final approval of the manuscript. PDL and FL: collection and assembly of the data and final approval of the manuscript. MV: statistical analysis and final approval of the manuscript. CPO and LZ: data analysis and interpretation and final approval of the manuscript. All authors contributed to the article and approved the submitted version.

## Conflict of Interest

The authors declare that the research was conducted in the absence of any commercial or financial relationships that could be construed as a potential conflict of interest.

## Footnotes

1 https://www.thermofisher.com/document-connect/document-connect.html?url=https%3A%2F%2Fassets.thermofisher.com%2FTFS-Assets%2FLSG%2Fbrochures%2FCO28730-Crt-Tech-note_FLR.pdf&title=Q3J0LCBhIHJlbGF0aXZlIHRocmVzaG9sZCBtZXRob2QgZm9yIHFQQ1IgZGF0YSBhbmFseXNpcyBvbiB0aGUgUXVhbnRTdHVkaW8mdHJhZGU7IDEySyBGbGV4IHN5c3RlbSB3aXRoIE9wZW5BcnJheSZyZWc7IHRlY2hub2xvZ3k=

2 https://biit.cs.ut.ee/clustvis/
